# 

**Published:** 1890-09

**Authors:** 


### LITERARY.

A NEW MAGAZINE.—The Polytechnic is the name of a new magazine to be published in Chicago, the initial number of which will be issued next month. Like the London magazine of that name, it will be the organ of a Polytechnic Institute, which in this case has been lately started in Chicago, and will be modelled after the famous London institute of similar name, an interesting account of which was given in the "Century" for June. The first number will be largely descriptive of the work of the Institute, especially its Trade Schools, a peculiar feature of which is that students may earn their expenses while in attendance, and can learn almost any trade. As this promises to solve the vexed apprenticeship question, all master associations are warm supporters of the movement. An article on the new Evening Medical College of Chicago is also included in this number. The ladies will be interested in the description of the cooking, millinery and dressmaking schools of the Chicago Polytechnic Institute. Published at the S. E. corner Madison street and Fifth avenue, Chicago, Ill. Sample copy, 10 cents.

The following treatises have been received, for which we thank the writers.

FIVE CASES OF VAGINAL HYSTORECTOMY ; by W. F. McNutt, M. D., L. R. C. P., Ed., etc.; Professor Principles and Practice of Medicine, University of California.

VARICOCELE ; by Thomas W. Kay, M. D., Ex-Surgeon to the Johanniter Hospital, at Beyraut, Syria, Scranton, Pa.

THE CENTURY for September, among many interesting articles, contains one entitled, "Finding Paths to California," upon which Gen. Fremont was engaged at the time of his death, and which has been completed by his widow. There is presented a fine portrait of "The Pathfinder," taken in '49. Another paper, "How California came into the Union," is a valuable contribution to history.

